# Infrared drying characteristics of locust (*L. migratoria*): physical and techno-functional characterization of locust powder

**DOI:** 10.1007/s13197-025-06340-w

**Published:** 2025-06-02

**Authors:** Işıl Barutçu Mazı, Bekir Gökçen Mazı, Hasan Sevgili

**Affiliations:** 1 Department of Food Engineering, Agricultural Faculty, Ordu University, Ordu, Turkey; 2 Department of Molecular Biology and Genetic, Faculty of Art and Science, Ordu University, Ordu, Turkey

**Keywords:** Edible insect, Locust powder, Infrared drying, Drying kinetics, Carr’s index, Water holding capacity

## Abstract

This study examined some physical and techno-functional properties of locust (*Locusta migratoria*) powder produced by infrared drying at temperatures of 60, 70, and 80 °C. Water activity, pH, Carr’s index, Hausner ratio, water holding capacity (WHC), oil holding capacity (OHC), and color parameters of the powders were measured. In addition, infrared drying kinetics were analyzed. Increasing the drying temperature from 60 to 80 °C resulted in a 2.8-fold reduction in drying time, without causing significant changes in the measured properties. Except for WHC, the properties of infrared-dried powders were comparable to those of freeze-dried powder. WHC (2.1–2.3 g water/g) of infrared-dried powders was lower than that of the freeze-dried powder (3.3 g water/g). The locust powder exhibited poor flowability. Drying occurred in the falling rate period, with the Page model best describing the drying behavior. Effective moisture diffusivity (*D*_eff_) ranged from 1.17 × 10^–10^ to 10.9 × 10^–7^ m^2^/s, and the activation energy was calculated as 53.81 kJ/mol. The influence of drying temperature on the drying rate was most pronounced during the early stages of drying. Overall, infrared drying, with its shorter processing time, appears to be a promising alternative for the production of locust powder.

## Introduction

There is a need for sustainable food sources due to the interconnected challenges including population growth, resource scarcity, environmental impact, climate change, biodiversity loss, food security. These challenges drive the necessity to balance food production demands with global health, well-being, and future generations' needs. Traditional food sources exert significant pressure on soil health and water resources and contribute to greenhouse gas emissions, making them increasingly unsustainable. This highlights the urgent need to develop sustainable alternatives that can meet nutritional demands without further harming the planet.

Among these alternatives, insects have been utilized as a protein source throughout history in various regions worldwide (Van Huis 2017). Many edible insect species are part of traditional diets, particularly in tropical regions (Omuse et al. 2024). In Asia, many parts of Africa, and Latin America, people consume insects either as snacks, or as a part of their daily diets (Menozzi et al. 2017; Omuse et al. 2024). Traditional cooking techniques for insects include steaming, roasting, smoking, frying, boiling, and drying (Melgar-Lalanne et al. 2019). Offering insects in powdered form enhances their acceptance as a food source by minimizing their distinct visual characteristics (Menozzi et al. 2017). Melgar-Lalanne et al. (2019) identified the main commercial forms of edible insects as flavored snacks, energy bars and powders. Insect powders are used in food product formulation or as starting materials for producing protein concentrates or isolates (Hernández-Álvarez et al. 2021). Several countries including the United States, Belgium, the United Kingdom, the Netherlands, France, Mexico, Germany, and Australia have commercially available insect-derived powdered products (Melgar-Lalanne et al. 2019). The demand for insect-based foods has been steadily increasing, albeit at varying rates across different regions and markets. Edible insects, as an affordable and environmentally friendly source of animal protein, are increasingly being labeled as the food of the future.

There are over 2000 edible insect species worldwide. Among these, those belonging to the order Orthoptera (grasshoppers, locusts, and crickets) hold a significant place (Melgar-Lalanne et al. 2019). *Locusta migratoria* (Linnaeus 1758) (migratory locust), a member of the Acrididae family (Ingrisch and Rentz 2009), has gained approval as novel food in the European Union market following safety assessments by the European Food Safety Authority (Liguori et al. 2022). Migratory locusts contain high levels of protein (58–66%), lipids (18–30%), unsaturated fatty acids, and essential minerals (Aguilera et al. 2021; Barutçu Mazı 2024; Fombong et al. 2021). Therefore, incorporation of migratory locust powder offers an innovative approach for enhancing the nutritional content of various food products.

Producing insect powder requires a drying process. In the drying of edible insects, various drying methods such as freeze drying, oven drying, microwave drying, fluidized bed drying, vacuum drying, sun drying and solar drying have been utilized (Hernández-Álvarez et al. 2021; Parniakov et al. 2022). The method used for drying plays a crucial role in determining the final quality of insect powder, including its physicochemical and techno-functional properties (Bawa et al. 2020; Fombong et al. 2017; Khatun et al. 2021; Kröncke et al. 2018; Lucas-González et al. 2019; Mishyna et al. 2021; Mugova et al. 2021). However, the same drying method may not yield identical results for all insect species, as the impact of drying on insect powder properties varies due to differences in the composition and structure of different species (Khatun et al. 2021; Mishyna et al. 2020, 2021; Nyangena et al. 2020). For instance, Mishyna et al. (2020) demonstrated that different drying methods had distinct effects on the volatile profiles of edible locusts and silkworms. Similarly, Khatun et al. (2021) reported that although *Acheta domesticus* and *Gryllus assimilis* are both cricket species, freeze drying and oven drying had varying effects on their physicochemical properties. Hot-air drying and freeze drying are among the most widely studied methods for insect dehydration, each offering distinct advantages and limitations (Hernández-Álvarez et al. 2021). Several studies have reported that hot-air drying yields similar proximate, mineral, fatty acid, and amino acid compositions in grasshoppers and crickets compared to freeze drying (Fombong et al. 2017; Khatun et al. 2021), while also resulting in lower fat oxidation in mealworms and crickets (Keil et al. 2022; Khatun et al. 2021; Kröncke et al. 2018). In contrast, freeze drying has been shown to enhance the techno-functional properties of cricket flours, including WHC, OHC, swelling capacity, and emulsion capacity compared to hot air drying (Lucas-González et al. 2019). Moreover, it produces lighter-colored insect powders, which may be desirable for certain food applications (Azzollini et al. 2016; Khatun et al. 2021; Lucas-González et al. 2019; Mishyna et al. 2020). However, despite its advantages in preserving nutritional profile, color, and sensory properties, freeze drying is less favorable for industrial use due to its high energy consumption, long drying times, and operational costs (Liu et al. 2022; Parniakov et al. 2022). Given these limitations, researchers have explored alternative drying methods, such as microwave drying, which offers advantages in terms of drying efficiency and product quality. Bawa et al. (2020) found that microwave-dried cricket powder had higher levels of mineral elements and vitamin B2, improved color parameters, and lower microbiological loads compared to oven-dried cricket powder.

Among alternative drying methods, infrared drying has also emerged as a promising drying technology for biological materials. Infrared drying has been extensively studied in the context of fruits, vegetables, meats, and grains, where it has demonstrated significant improvements in drying efficiency and product quality (Delfiya et al. 2022; Huang et al. 2021). Notably, Aykın Dinçer (2023) proposed infrared drying as one of the effective drying methods for meat products due to its ability to enhance drying rate while maintaining desirable textural and nutritional attributes. Compared to conventional convective heat drying, IR drying offers advantages such as reduced drying time, enhanced energy efficiency, and better retention of sensory and nutritional properties (Huang et al. 2021). The mechanism of infrared radiation facilitates faster water removal by directly transferring energy to the material’s surface and subsurface layers, thereby accelerating drying kinetics (Delfiya et al. 2022). These improvements in drying kinetics are particularly important, as they directly influence processing time, energy consumption, and the preservation of heat-sensitive compounds. Furthermore, studies have shown that drying parameters such as radiation intensity, distance from the infrared source, and drying temperature significantly affect the drying characteristics and final quality of food products (Delfiya et al. 2022; Huang et al. 2021). Despite the well-documented benefits of infrared drying in a wide range of food systems, research specifically addressing its application to insect powders remains limited. Khampakool et al. (2020) examined the effects of infrared-assisted freeze drying on *Protaetia brevitarsis* larvae. They reported improved drying kinetics, reduced hardness, and better preservation of glutamic acid and proline compared to hot-air drying. Keil et al. (2022) compared several drying methods, including freeze drying, microwave drying, infrared drying, oven drying, and high-frequency drying for yellow mealworm larvae. Infrared drying was more cost-effective than microwave drying and resulted in lower lipid oxidation than freeze drying. Overall, these studies support the potential of infrared drying as an efficient technique for insect dehydration. Given its potential to enhance both efficiency and quality, further investigation into the effects of infrared drying on drying kinetics and quality properties of insect-based powders is warranted.

While numerous studies have investigated the effects of different drying methods on various insect species, the literature shows that drying outcomes can vary significantly depending on species-specific composition and structural characteristics. Additionally, although infrared drying has been extensively explored in other food systems and has shown promise in insect dehydration, current studies involving insects have been limited in scope. To the best of our knowledge, no previous research has specifically examined the effects of infrared drying on the drying kinetics, physicochemical, and techno-functional properties of locust powder. By addressing this gap, the present study contributes novel insights into the applicability and performance of infrared drying for *L. migratoria*, thus advancing the understanding of species-specific drying behavior and supporting future industrial applications. Therefore, this study aimed to fill that gap by investigating water activity, pH, Carr’s index, Hausner ratio, water holding capacity (WHC), oil holding capacity (OHC), and color parameters of locust powder produced using infrared drying. This study also aimed to study the drying kinetics of locusts at temperatures of 60, 70, and 80 °C and model the data using thin-layer drying models.

## Material and methods

### Materials

Live locusts (*L. migratoria*) were acquired from a commercial insect farm (Mira Co., Aksu, Antalya, Türkiye) and transported to the laboratory. Upon arrival, the insects were euthanized using 100% CO_2_ (≥ 99.9% purity) following established insect handling protocols (Singh et al. 2020). The euthanasia process was conducted in a sealed chamber into which CO₂ was gradually introduced to ensure controlled atmospheric displacement and to minimize insect stress. Following euthanasia, the locusts underwent pasteurization in water (90 °C, 10 min) and were later stored at − 18 °C until further use.

### Drying process

Locust samples were dried by infrared moisture analyzer (MAC 50, Radwag, Poland). The IR emiter heating module (400 W), equipped with balance, was set to a standard drying profile. Approximately 20 g of samples were spread evenly over the pan and dried until the moisture content of the samples reached 5% (w.b.). To investigate the impact of drying temperature on the drying rate of locust samples, drying experiments were carried out at temperatures of 60, 70, and 80 °C (Bawa et al. 2020). After drying, samples were ground with a coffee grinder (PRG 259, Premier, Istanbul, Türkiye). The ground samples were then passed through a Mesh #40 sieve, corresponding to a pore size of approximately 415 μm, before being used in subsequent analyses.

The properties of infrared-dried locust powders were compared with that of locust powder produced using freeze drying in a lab-scale freeze dryer (FreeZone 2.5L 7670530, Labconco) at − 50 °C and 0.1 mbar vacuum pressure.

### Drying kinetics

The average moisture content of locusts at the beginning of drying process was calculated as 76.70% (w.b.) based on the weight loss determined by oven drying at 105 °C until a constant weight was reached.

During infrared drying, the weight of the samples was recorded at 5 s intervals. The moisture content of the samples at any given time during drying was calculated using the recorded weight and dry matter values. All experiments were conducted in triplicate, and the average moisture content values were reported. The drying rates (R) (kg water/kg d.b. min), were calculated using Eq. 1.

1 $$R = \frac{{(X_{t} - X_{t + \Delta t} )}}{{\left( {\Delta t} \right)}}$$

where X_t_ is the moisture content (kg water/kg d.b.) at time t (min), X_t+Δt_ is the moisture content (kg water/kg d.b.) at time t + Δt (min).

Dimensionless moisture ratio (MR) values were determined using the following equation:

2 $$MR = \frac{{X_{{\mathrm{t}}} - X_{e} }}{{X_{0} - X_{e} }}$$

where X_o_ is the initial moisture content (kg water/kg d.b.) and X_e_ is the equilibrium moisture content (kg water/kg d.b.). The equilibrium moisture content was determined using the final moisture content at the end of the drying process.

The experimental MR data were applied to four commonly employed thin-layer drying models (Newton, Page, Henderson and Pabis, and logarithmic), as listed in Table 1. The Newton and Page models are based on Newton’s law of cooling, while the Henderson and Pabis and logarithmic models are derived from Fick’s second law of diffusion (Ertekin and Firat 2017). These models have been widely used in the food drying literature due to their simplicity and proven effectiveness in representing moisture loss in agricultural products (Ertekin and Firat 2017). Furthermore, these models have been successfully employed to describe the drying behavior of high-protein food matrices such as meat products, including applications involving microwave and infrared drying (Elmas et al. 2020; Kamiloğlu et al. 2023; Ozyalcin et al. 2023). Although studies on insect drying kinetics are limited, existing research has used the Page and Newton models with good fitting performance (Azzollini et al. 2016; Khampakool et al. 2020).

**Table 1:** Thin-layer drying models

Model	Equation
Newton	MR = exp (− kt)
Page	MR = exp (− kt^n^)
Henderson and Pabis	MR = a exp (− kt)
Logarithmic	MR = a exp (− kt) + c

Parameters of models were predicted by nonlinear least-squares procedure applied by MATLAB software (R2021b, Mathwork, Inc., MA, US). To determine the best fitted model, the determination coefficient (R^2^), the sum of square error (SSE), the root mean square error (RMSE) values were calculated using Eqs. 3, 4, and 5, respectively.

3 $$R^{2} = 1 - \left[ {\frac{{\mathop \sum \nolimits_{i = 1}^{N} \left( {MR_{exp, i} - MR_{pred, i} } \right)^{{2}} }}{{\mathop \sum \nolimits_{i = 1}^{N} \left( {MR_{exp, i} - MR_{mean} } \right)^{{2}} }}} \right]$$

4 $$SSE = \mathop \sum \limits_{i = 1}^{N} \left( {MR_{exp, i} - MR_{pred, i} } \right)^{{2}}$$

5 $${\mathrm{RMSE}} = \left[ {\frac{{{\mathrm{SSE}}}}{{\upnu }}} \right]^{{\frac{{1}}{{2}}}} \quad {\mathrm{where}}\;\nu = N - m$$

where MR_exp,i_ and MR_pred,i_ represent experimental and predicted MR values, respectively; MR_mean_ is the mean of experimental MR values; N is the total number of experiments; m is the number of constants in the model.

The migration of moisture and/or vapor during the falling rate period is controlled by diffusion. Fick's second law of diffusion was used to determine the effective diffusivity values. Locusts were considered as infinite cylinders. By assuming negligible shrinkage and external resistance, a uniform initial moisture distribution, a constant effective diffusion coefficient (D_eff_), and one-dimensional diffusion in the radial direction, the mathematical solution of the Fickian equation for an infinite cylinder, given in Eq. 6, was used.

6 $$MR = \mathop \sum \limits_{n = 1}^{\infty } \frac{4}{{{\uplambda }_{n}^{2} }}exp\left( { - \frac{{{\uplambda }_{n}^{2} D_{eff } t}}{{r^{2} }}} \right)$$

where λ_n_ represents the roots of Bessel function of zero order (λ_1_ = 2.405), t is the experimental drying time (s) and r is the radius of the cylinder, n is the positive integer, D_eff_ is the effective moisture diffusivity (m^2^/s). For long drying times, the first term of a series solution is considered, and equation could be simplified into following equation (Eq. 7) (Brennan 1994).

7 $$MR = \frac{4}{{{\uplambda }_{1}^{2} }}exp\left( { - \frac{{{\uplambda }_{n}^{2} D_{eff } t}}{{r^{2} }}} \right)$$

Equation 7 may be written in logarithmic form as follows.

8 $$\ln \left( {MR} \right) = - 0.369 - 5.784\left( {\frac{{D_{eff } t}}{{r^{2} }}} \right)$$

The slope is calculated by plotting ln MR versus time. D_eff_ is determined from the slope using Eq. 9.

9 $$D_{eff } = - \left( {\frac{{slope. r^{2} }}{5.784}} \right)$$

Activation energy (E_a_) was calculated using the following Arrhenius type equation (Eq. 10)

10 $$D_{eff } = D_{0} exp\left( { - \frac{{E_{a } }}{RT}} \right)$$

where D_o_ is the Arrhenius factor, E_a_ is the activation energy (J/mol), R is the universal gas constant (8.314 J/mol·K), and T is the absolute temperature (K).

### Water activity

Water activity was measured using a thermo-hygrometer (Testo 645, Testo GmbH & Co., Lenzkirch, Germany) equipped with a hermetically sealed measurement chamber at room temperature (23 ± 2 °C).

### Color parameters

The L* (lightness), + a* (redness-greenness), and + b* (yellowness-blueness) color parameters of the locust powder were determined using a colorimeter (CSM1, PCE, Germany) with illuminant D65, 4 mm aperture. The sample's color value was determined by averaging the L*, a*, and b* readings taken from ten distinct positions on its surface. Each drying treatment was replicated ten times, and the results were averaged. The chroma (c) values and hue angle (h) of powder were calculated using the following equations:

11 $${\mathrm{Hue}} = {\text{arctan }}\left( {b*/a*} \right),\quad a*, b*> 0$$

12 $${\mathrm{Chroma}} = \sqrt {\left( {a*} \right)^{2} + (b*)^{2} }$$

### pH

A 200 mg sample was mixed with 19.8 mL of deionized water, and its pH was measured at room temperature using a pH meter (Ohaus starter 3100, USA) (Morr et al. 1985).

### Bulk density, Carr’s index (CI) and Hausner ratio (HR)

Two grams of locust powder were weighed into a measuring cylinder without allowing contact with the inner walls. The volume occupied by the powder was recorded for calculating the poured bulk density (ρ_p_) (g/mL). Subsequently, the same cylinder was raised to a height of 15 cm and tapped onto a flat surface. The volume was recorded, and the process was repeated until the difference between the last two readings became negligible. Using the final recorded volume, the tapped density (ρ_b_) (g/mL) was calculated (Farahmandfar et al. 2019).

Carr’s index (CI) (Carr 1965) and Hausner ratio (HR) (Hausner 1967) values were calculated using Eqs. 13, and 14, respectively.

13 $$CI = \frac{{\rho_{t} - \rho_{p} }}{{\rho_{t} }} \times 100$$

14 $$HR = \frac{{\rho_{t} }}{{\rho_{p} }}$$

### Water holding capacity (WHC)

A 0.5 g powder sample was hydrated in 30 mL of distilled water for 24 h, followed by centrifugation at 2000 × g for 25 min. Subsequently, the supernatant was removed, and the tubes were weighed. The WHC was calculated using the following formula (Romdhane et al. 2015):

15 $${\mathrm{WHC}}\,({\mathrm{g}}\;{\mathrm{water/g}}\;{\mathrm{sample}}) = ({\mathrm{W}}_{{2}} - {\mathrm{W}}_{{1}} )/{\mathrm{W}}_{{1}}$$

where W_1_ (g) is the initial weight of sample and W_2_ (g) is the final weight of sample.

### Oil holding capacity (OHC)

A 0.5 g powder sample was added to 10 mL of sunflower oil and subsequently centrifuged at 2000 × g for 20 min. Afterward, the supernatant was removed, and the tubes were weighed. The OHC was calculated using the following formula (Romdhane et al. 2015):

16 $${\mathrm{OHC}}\;\left( {{\mathrm{g}}\;{\mathrm{oil/g}}\;{\mathrm{sample}}} \right) = ({\mathrm{W}}_{{2}} - {\mathrm{W}}_{{1}} )/{\mathrm{W}}_{{1}}$$

where W_1_ (g) is the initial weight of sample and W_2_ (g) is the final weight of sample.

### Statistical analysis

Each experiment was replicated three times, unless stated otherwise, and the results were expressed as means ± standard deviations. Mean comparisons were conducted through analysis of variance (ANOVA) at a significance level of 5% with Minitab 17 software. Subsequent multiple comparisons were performed using Post-hoc analysis with Tukey’s test.

## Results and discussion

### Drying process

**Fig. 1:**
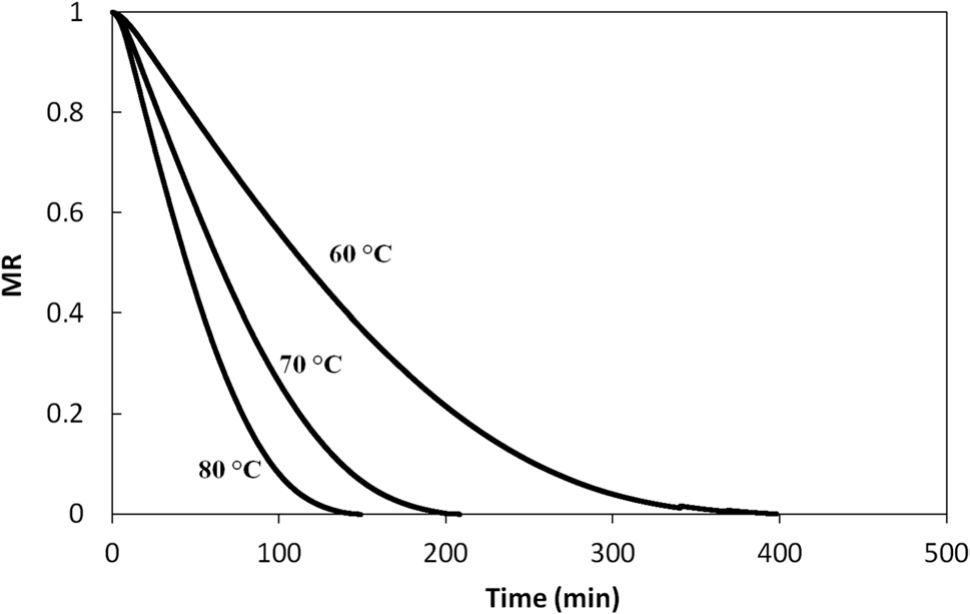
Moisture ratio (MR) versus drying time

The infrared drying curves of locusts, illustrating the relationship between moisture ratio and drying time at different temperatures, are presented in Fig. 1. To enable a fair comparison of the physicochemical and techno-functional properties of the resulting powders, all samples were dried to a final moisture content of approximately 5% (w.b.). The time required to reduce the initial moisture content of about 76.70% (w.b.) to this final level was 349, 180, and 126 min at 60, 70, and 80 °C, respectively. Increasing the drying temperature from 60 to 80 °C resulted in a substantial 2.8-fold decrease in drying time.

**Fig. 2:**
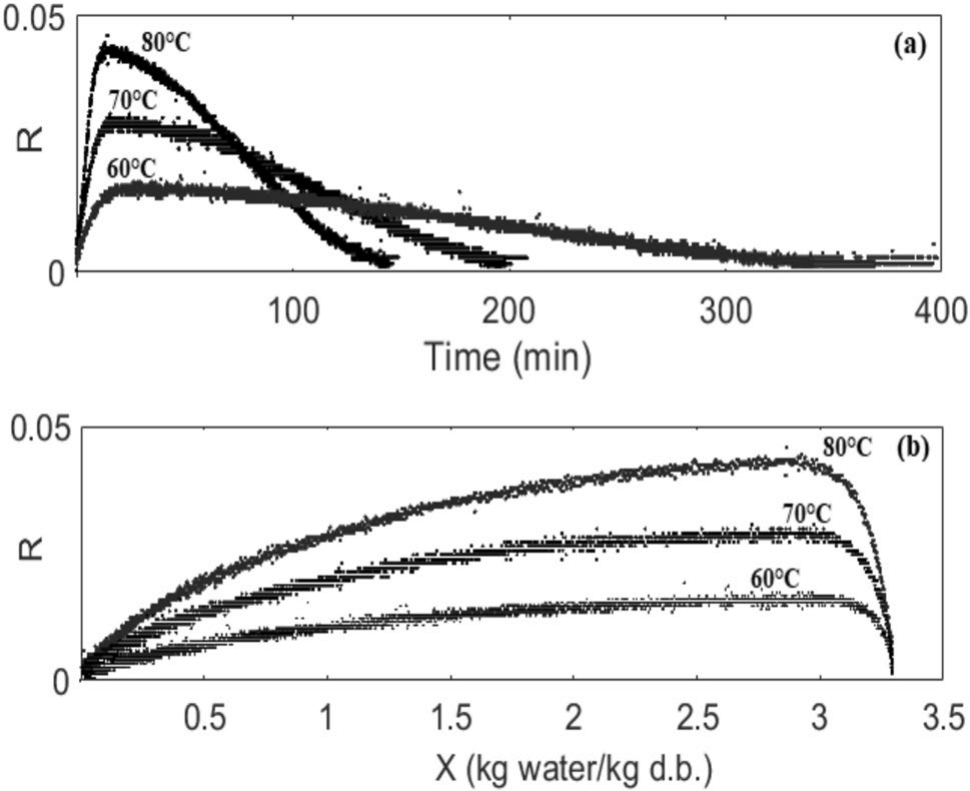
Drying rate (R) versus drying time (**a**) and drying rate (R) versus free moisture content (**b**) curves for different infrared drying temperatures

Drying rate curves of locusts at different temperatures are shown in Fig. 2. All curves exhibited an initial adjustment phase, during which the solid surface comes to equilibrium with the surrounding air. At 80 °C, the drying rate declined rapidly following this initial phase, indicating the absence of a constant rate period. Even at lower temperatures, no distinct constant drying rate period was observed. This suggests that diffusion dominated the moisture movement within the locust samples during infrared drying. These findings are consistent with those reported by Álvarez et al. (2021) for beef drying. However, literature on the drying behavior of edible insects remains limited. In a related study, Azzollini et al. (2016) found that oven drying of mealworm larvae occurred exclusively within the falling rate period.

As expected, at similar average free moisture contents, the drying rate increased with rising temperature. The maximum drying rates observed at 60, 70 and 80 °C were 0.017, 0.03, and 0.043 kg water/kg d.b. min, respectively. Elevated temperatures enhance the rate of heat transfer, thereby accelerating the movement of water molecules from within the tissue to the surface. Moisture migration in locust tissue may also be influenced by microstructural changes resulting from protein denaturation during drying. Protein denaturation reduces the water-binding capacity of the protein matrix and leads to the formation of moisture channels associated with tissue shrinkage. The structural changes, such as shrinkage and the development of voids, are dependent on both the drying temperature and duration. The greater presence of voids at higher drying temperatures may facilitate faster moisture removal, contributing to the observed increase in drying rate. Throughout the drying process, drying rates declined progressively with the reduction in free moisture content. After approximately 70 min of drying, the drying rates at 70 and 80 °C converged to a similar value of about 0.025 kg water/kg d.b. min. Beyond this point, the samples dried at 80 °C exhibited a slower moisture loss compared to those dried at 70 °C. Similarly, after around 140 min, the drying rate at 60 °C surpassed that at 70 °C.

### Drying kinetics

The average moisture ratio (MR) values were fitted to the drying models given in Table 1, and the corresponding model coefficients and statistical parameters are summarized in Table 2. All models exhibited a good fit to the experimental data. The R^2^_adj_, SSE, and RMSE values ranged from 0.950 to 0.999, 0.235 to 18.9, and 0.011 to 0.072, respectively. Among the models evaluated, the Page model was identified as the most suitable for describing the thin-layer drying behavior of locusts, as it consistently produced the lowest SSE and RMSE values and the highest R^2^ values across all drying conditions. The drying rate constant (k) derived from Page model, which reflects the rate of moisture removal from the sample, ranged between 0.0007 and 0.0021 1/min. For all models, the k value increased linearly (R^2^ ≥ 0.99) with increasing temperature. A similar trend was reported by Azzollini et al. (2016) during the oven drying (50–70 °C) of *Tenebrio molitor* larvae, where the Page model successfully described the drying kinetics, and k values increased with temperature, ranging from 0.0075 to 0.0331 1/min. In another study, Khampakool et al. (2020) applied the Page model to describe the drying behavior of *Protaetia brevitarsis* larvae under infrared-assisted freeze-drying and noted variable k values (0.00000012–0.007032 1/min), depending on the specific process parameters. The parameter n in the Page equation is related to the material’s microstructure and the nature of moisture diffusion (pure diffusion (*n* = 1), sub-diffusion (*n* < 1) and super-diffusion (*n* > 1) (Simpson et al. 2017). In the present study, the n values were consistently greater than 1, indicating a super-diffusive behavior.

**Table 2:** Fitting parameters of the thin layer drying models

Model	Drying temperature	SSE	Adj R^2^	RMSE	Parameters			
					k	n	a	c
Newton	60 °C	18.900	0.959	0.063	0.0072			
	70 °C	12.348	0.951	0.070	0.0130			
	80 °C	9.216	0.950	0.072	0.0187			
Page	60 °C	0.900	0.998	0.014	0.0007	1.450		
	70 °C	0.426	0.998	0.013	0.0013	1.513		
	80 °C	0.235	0.999	0.011	0.0021	1.531		
Henderson&Pabis	60 °C	11.000	0.977	0.047	0.0082		1.143	
	70 °C	6.654	0.974	0.052	0.0150		1.160	
	80 °C	4.737	0.974	0.051	0.0217		1.169	
Logarithmic	60 °C	2.100	0.995	0.021	0.0056		1.256	− 0.179
	70 °C	1.260	0.995	0.022	0.0096		1.310	− 0.224
	80 °C	1.058	0.994	0.024	0.0145		1.298	− 0.199

The average D_eff_ values for locusts ranged from 2.83 × 10^–10^ to 8.49 × 10^–10^ m^2^/s (Table 3). D_eff_ values followed a trend similar to that of the k values. D_eff_ values increased progressively with increasing temperature. The enhancement in Deff at higher temperatures may be attributed to the increased vapor pressure within the sample, which facilitates faster moisture migration through the tissue. Activation energy (E_a_) is the energy required to activate moisture diffusion within the sample. It is a measure of the temperature sensitivity of D_eff_. A higher E_a_ value implies a greater dependence of diffusivity on temperature. In this study, the activation energy was calculated as 53.81 kJ/mol. For various plant-based food materials, including fruits, vegetables, and nuts, subjected to infrared/infrared assisted drying, reported D_eff_ values typically range between 1.17 × 10^–10^ and 10.9 × 10^–7^ m^2^/s, while E_a_ values vary between 11.18 and 110 kJ/mol (Delfiya et al. 2022). D_eff_ and E_a_ values for edible insects and meat products dried by different methods have also been reported by several researchers (Table 3). The values obtained in this study are within the range of values given in literature.

**Table 3:** Effective diffusivity (D_eff_) and activation energy (E_a_) values of various insect and meat products dried under different drying conditions

Food	Drying method	D_eff_ × 10^10^ (m^2^/s)	E_a_	References
Locust powder	Infrared drying			Present study
	60 °C	2.83 (R^2^ = 0.946)	53.81 (R^2^ = 0.982) kJ/mol	
	70 °C	5.67 (R^2^** = **0.948)		
	80 °C	8.49 (R^2^** = **0.955)		
*Protaetia brevitarsis* larvae	Infrared assisted freeze-drying (vacuum of 6.67 Pa, infrared power intensity of 5.0 kW/m^2^)	2.41–18.87	–	Khampakool et al. (2020)
*Tenebrio molitor* larvae	Forced air oven drying (50–70 °C)	0.45–1.62	52.1 kJ/mol	Azzollini et al. (2016)
Beef	Convective drying (CD) (60–80 °C); microwave drying (MD) (487–889 W)	CD: 2.02–7.61 MD: 2.26–3.73	64.15 kJ/mol	Kamiloğlu et al. (2023)
Turkey breast meat	Hot air drying (AD) (60–90 °C); microwave drying (MD) (180–540 W); freeze drying (0.1–0.2 mbar)	AD: 2.03–2.53 MD: 27.89–103.96 FD: 2.99–3.36	HA: 7.48 kJ/mol MD: 6.043 W/g	Elmas et al. (2020)
Seabass	Oven drying (OD) (60–80 °C); infrared drying (ID) (60–80 °C); microwave drying (MD) (140–350 W)	OD: 4.96–8.02 ID: 5–11.1 MD: 94.2–324	OD: 23.42 kJ/mol ID: 39.05 kJ/mol MD: 0.96 W/g	Ozyalcin et al. (2023)

### Water activity (a_w_) and pH

Water activity (a_w_), defined as the amount of free, unbound water available in a system, is a critical factor influencing the quality, stability, and functional properties of powdered food products (Derossi et al. 2011; Rockland and Stewart 2013). It plays a key role in determining the susceptibility of food powders to microbial growth and chemical reactions (e.g., lipid oxidation and Maillard browning) (Derossi et al. 2011; Rockland and Stewart 2013). In addition to its impact on microbial and chemical stability, water activity also affects the physical behavior of powders, particularly their bulk flow properties (Chang et al. 1998). At a_w_ < 0.20, water is tightly bound and does not affect flow; at a_w_ > 0.60, free water may form liquid bridges, impairing powder flow (Suhag et al. 2024). Water activity values of the locust powders are presented in Table 4, ranging from 0.490 to 0.529. The minimum water activity required to prevent microbial growth is reported as 0.6 for foods (López-Malo and Alzamora 2015). Since the water activity values of the locust powders were below this threshold, microbial proliferation is unlikely, contributing to the microbiological safety and storage stability of the product. Bawa et al. (2020) reported water activity values of 0.40 and 0.38 for oven-dried and microwave-dried *Acheta domesticus* powders, respectively, at similar moisture levels (~ 5%). While these values are slightly lower than those obtained in the present study, the differences may be attributed to species variation, drying parameters, or measurement conditions. Khatun et al. (2021) found varying water activity values depending on both drying method and insect species. For *A. domesticus*, freeze-dried and oven-dried samples showed a_w_ values of 0.26 and 0.31, respectively, whereas for *G. assimilis*, the corresponding values were 0.32 and 0.27. In addition, locust powder is known to have a relatively high fat content, which increases its susceptibility to lipid oxidation during storage. The relationship between water activity and lipid oxidation is known to be non-linear and complex. The oxidation rate tends to decrease as a_w_ increases up to 0.2, then rises with increasing aw until around 0.75, after which it declines again (Derossi et al. 2011). Given that the a_w_ values of the locust powders in this study fall within the intermediate range (~ 5), oxidative stability could be a concern during extended storage. The water activity values were relatively similar across the infrared-dried and freeze-dried samples in our study. Similarly, Lucas-González et al. (2019) observed no significant difference in water activity between freeze-dried and oven-dried cricket powders, supporting the observation that drying method may not always have a substantial impact on aw values.

**Table 4:** Physicochemical and techno-functional properties of locust powder

Property	Freeze drying	Infrared drying		
		60 °C	70 °C	80 °C
pH	4.78 ± 0.03 b	5.66 ± 0.06 a	5.66 ± 0.06 a	5.61 ± 0.04 a
a_w_	0.500 ± 0.002 c	0.529 ± 0.001 a	0.511 ± 0.003 b	0.490 ± 0.004 d
ρ_p_ (g/ml)	0.294 ± 0.017 a	0.296 ± 0.011 a	0.302 ± 0.010 a	0.302 ± 0.009 a
ρ_t_ (g/ml)	0.528 ± 0.001 a	0.518 ± 0.015 ab	0.505 ± 0.007 b	0.528 ± 0.000 a
Hausner ratio	1.798 ± 0.106 a	1.750 ± 0.039 a	1.672 ± 0.030 a	1.746 ± 0.055 a
Carr’s index, %	44.260 ± 3.350 a	42.838 ± 1.290 a	40.179 ± 1.062 a	42.680 ± 1.820 a
Color parameters				
L*	30.35 ± 1.24 b	32.45 ± 1.43 a	32.26 ± 1.06 a	31.29 ± 0.98 ab
a*	9.38 ± 0.28 c	9.33 ± 0.28 c	9.82 ± 0.46 b	10.24 ± 0.38 a
b*	11.62 ± 0.47 a	12.23 ± 0.87 a	12.41 ± 0.86 a	12.09 ± 0.69 a
c	14.93 ± 0.50 b	15.39 ± 0.85 ab	15.83 ± 0.94 ab	15.85 ± 0.70 a
h	51.06 ± 0.85 b	52.59 ± 1.24 a	51.60 ± 0.94 ab	49.68 ± 1.32 c
WHC (g water/g sample)	3.349 ± 0.025 a	2.266 ± 0.106 b	2.192 ± 0.087 b	2.075 ± 0.240 b
OHC (g oil/g sample)	1.584 ± 0.286 a	1.707 ± 0.062 a	1.618 ± 0.064 a	1.683 ± 0.047 a

ρ_p_: poured bulk density; ρ_t_: tapped bulk density; c: chroma; h: hue angle

*Values represent mean ± standard deviation. Different small letters in the same row shows significant difference (*p* < .05).

The natural pH of insect powder plays a critical role in its compatibility and effectiveness in different food systems. The pH values of the locust powders ranged from 4.78 to 5.66 (Table 4). The infrared drying temperature had no significant effect on the pH of the powders. However, the sample subjected to freeze-drying exhibited a lower pH value compared to those dried using infrared. This difference may be attributed to the retention of free acidic groups during the freeze-drying process, which is known to better preserve the chemical integrity of heat-sensitive compounds. While the drying method influenced the pH in the present study, Lucas-González et al. (2019) reported no statistically significant differences in pH between oven-dried (6.31) and freeze-dried (6.48) house crickets. The pH values of the locust powders in our study were lower than those reported for most commercial plant protein isolates (6.0–7.7) (Jakobson et al. 2023) and sweet whey powders (6.02–6.58) (Alsaed et al. 2013), suggesting a relatively more acidic nature, which may influence their behavior in food systems.

### Color properties

Color is one of the major factors influencing sensory perception and consumer acceptance of foods (Hutchings 1999). The color of locust powder can affect the visual appeal of the final product when used as an ingredient in food formulations. The a* and b* values of the locust powders ranged from 9.33 to 10.24 and from 11.62 to 12.41, respectively (Table 4). During the processing of locust powder, enzymatic and non-enzymatic browning, as well as oxidative reactions, play a role in color changes (Lucas-González et al. 2019; Mishyna et al. 2020). In the present study, locusts were blanched prior to drying, which may have inhibited enzymatic browning by inactivating browning enzymes (Singh et al. 2020). Infrared drying resulted in b* values similar to that obtained by freeze drying (p < 0.05). This finding is consistent with the results of Khatun et al. (2021), who reported comparable b* values for freeze-dried and oven-dried house crickets, as well as with those of Azzollini et al. (2016), who obtained similar b* values for freeze-dried and oven-dried (50–70 °C) yellow mealworm larvae. However, our findings differ from those of Mishyna et al. (2020), who reported higher yellowness (b* value) in freeze dried (48 h) locust powder compared to the oven- and microwave-dried samples. Differences in b* values may be attributed to the degradation of color compounds during drying. *L. migratoria* contains various carotenoids, such as β-carotene, β-cryptoxanthin, α-carotene, lutein, zeaxanthin, and astaxanthin, depending on its diet (Goodwin 1949). According to Goodwin (1949), trans-β-carotene stands out as the primary carotenoid in locusts. Blanching may cause some loss of carotenoid pigments through leaching. During the subsequent drying step, carotenoids may undergo oxidation and isomerization reactions, the extend of which depends on factors such as heat, light, oxygen, and the presence of catalysts. Oxidation or isomerization of β-carotene and astaxanthin can result in pigment degradation, leading to a decrease in the b* color value of locust powder (Niamnuy et al. 2008). During infrared drying, heat may promote the isomerization of trans-carotenoids into their cis-forms. Although heat treatment during infrared drying can lead to carotenoid degradation, prolonged drying times in freeze-drying may also promote oxidative damage to these sensitive pigments (Dutta et al. 2005). In this study, infrared drying at 60, 70 and 80 °C took 5.8, 3.0, and 2.1 h, respectively, whereas freeze drying required 72 h. The similar b* values observed in freeze-dried and infrared-dried powders highlight the importance of drying duration in preserving color pigments. Supporting this, Bawa et al. (2020) reported significantly higher b* values for microwave-dried (at 600 W for 10 min) cricket powder compared to oven-dried (at 80 °C for 240 min) powder, despite the higher internal temperatures (up to 161 °C in 10 min) reached during microwave drying.

The freeze-dried powder exhibited a lower a* value compared to the infrared-dried powders at 70 and 80 °C. Similarly, previous studies have reported lower a* values in freeze-dried edible insect powders compared to those dried using oven or microwave drying methods (Azzollini et al. 2016; Khatun et al. 2021; Mishyna et al. 2020). The higher a* values observed in infrared-dried powders may be attributed, at least in part, to the occurrence of non-enzymatic browning reactions. In this study, the highest a* value was recorded at 80 °C, highlighting the role of elevated temperature in enhancing non-enzymatic browning. This finding aligns with previous work by Mishyna et al. (2020) and Bawa et al. (2020), who reported increased a* values in microwave-dried locust and cricket powders compared to oven-dried samples. Regarding L*, b*, and chroma values, no statistically significant differences were observed among the infrared dried samples. The L* values of infrared dried locust powders ranged from 31.29 to 32.45. The increase in temperature from 60 to 80 °C resulted in a slight, statistically insignificant decrease in L* value. A lower L* value indicates a darker color, which may result from intensified non-enzymatic browning at higher temperatures during infrared drying. The freeze-dried powder exhibited a slightly lower L* value (30.35) than the infrared-dried samples at 60 and 70 °C. This finding contrasts with those of Khatun et al. (2021) and Lucas González et al. (2019), who reported higher L* values for freeze-dried house crickets compared to oven-dried ones. However, it is consistent with observations by Mishyna et al. (2020), who found similar L* values (ranging from 38.6 to 42.8) among locust powders dried using freeze-drying (48 h), oven drying (at 60 °C for 24 h), and microwave drying (at 700 W for 13 min). In general, freeze-drying is known to yield higher lightness (L* value) in food products compared to other drying methods, primarily due to its ability to minimize non-enzymatic browning reactions (Khatun et al. 2021; Liu et al. 2022). Nevertheless, it is important to note that color changes during processing are influenced by multiple factors. One such factor may be differences in particle size distribution between freeze-dried and infrared-dried samples, which can affect light reflection and scattering, thereby altering the perceived color (Hutchings 1999).

### Bulk density, Carr’s index (CI) and Hausner ratio (HR)

The poured (ρ_p_) and tapped (ρ_t_) bulk densities of the locust powders are presented in Table 4. Bulk density reflects the interparticle voids in a sample and is a critical parameter affecting the handling, packaging, and storage of food powder (Koç et al. 2021). Higher bulk density allows for more efficient packaging and reduces transportation costs. It influences flowability and compressibility, which are essential for food processing operations (Shenoy et al. 2015; Suhag et al. 2024). Additionally, bulk density is an important factor affecting the storage stability of powders. Lower bulk density indicates more entrapped air within the void spaces, which increases oxygen exposure, accelerates oxidation, and ultimately shortens the shelf life of the product (Koç et al. 2021). In the present study, the poured and tapped densities of locust powders ranged from 0.294 to 0.302 g/ml and from 0.505 to 0.528 g/ml, respectively. These values align with previous findings. For instance, Aguilera et al. (2021) reported bulk densities between 0.15 and 0.52 g/ml for flours obtained from freeze-dried insect species (mealworm beetle, caterpillar, ant, locust, and cricket) with particle sizes below 0.5 mm. Specifically, the bulk density of locust flour reported by Aguilera et al. (2021) (0.15 g/ml) was lower than that found in the current study, which may be attributed to differences in sample-specific characteristics such as particle size, composition, and measurement methodology. Although limited studies are available, existing researches suggest that drying techniques can influence the functional properties of insect-based flours. For example, Mugova et al. (2021) found that sun dried flour from the armoured cricket (*A. discoidalis*), a key edible insect in Zimbabwe, exhibited lower bulk density than oven-dried flour. However, in the present study, different drying treatments did not exert a significant impact on bulk density values.

The Hausner ratio (HR) and Carr’s index (CI) are widely used indicators of powder flowability (Saker et al. 2019). The flow behavior of powdered food products plays a crucial role in ensuring efficiency and consistency during production, packaging, storage, and transportation (Ganesan et al. 2008). These flow indices are particularly important in high-throughput industrial settings, where consistent material behavior is essential. Poor flow characteristics can lead to challenges during dosing, filling, and packaging, ultimately affecting production efficiency (Suhag et al. 2024). Moreover, flowability affects the homogeneity of powder blends, which is critical for maintaining product quality (Cuq et al. 2013; Shenoy et al. 2015). HR is commonly interpreted as an indicator of powder cohesiveness (Lumay et al. 2012). According to Hausner (1967), a powder is considered to have low cohesiveness when HR < 1.2, intermediate when 1.2 < HR < 1.4, and high when HR > 1.4. Similarly, CI reflects powder flowability, with values interpreted as follow: very good (CR < 15), good (15 < CR < 20), fair (20 < CR < 35), poor (35 < CR < 45%) and very poor (CR > 45) (Carr 1965). In the present study, HR and CI values of locust powders ranged from 1.672 to 1.798 and 40.179 to 44.260%, respectively. These results indicate high cohesiveness and poor flowability, suggesting that the particles tend to agglomerate or interlock, which restricts smooth flow. This behavior may be partly attributed to the high protein and fat content of the locust powder (Chang et al. 1998). According to a previous study by Barutçu Mazı (2024), the fourth instar *Locusta migratoria* individuals, also utilized in the current study, were found to contain 63.8% protein and 11.0% fat based on proximate composition analysis. Son et al. (2019) reported that the flowability of full-fat mealworm powder (fat content: 37.6%) could not be assessed due to its extreme cohesiveness. Similarly, Koç et al. (2012) observed high HR (1.67–1.81) and CI (40.0–44.8%) values in spray-dried egg powder containing 51% protein. Flowability characteristics are influenced by various factors, including water activity and particle-related properties such as size, shape, distribution (Chang et al. 1998; Suhag et al. 2024). These parameters, in turn, are known to be affected by drying methods and processing conditions. In the current study, all locust powders had similar water activity levels (approximately 0.5) and the applied drying treatments did not exert a significant impact on flowability indices. Although freeze-dried powders showed slightly higher HR and CI values than those subjected to infrared drying, the differences were not statistically significant.

### Water holding capacity (WHC) and oil holding capacity (OHC)

WHC and OHC are important functional properties that influence the texture, mouthfeel, and stability of food products (Rajpurohit et al. 2025). WHC reflects a material’s ability to retain water, which contributes to moisture retention, juiciness, and shelf-life, particularly in baked goods, meat analogues, and rehydrated foods (Hong et al. 2022; Rajpurohit et al. 2025). OHC indicates the ability to absorb and retain oil, which is critical for flavor retention, improving the mouthfeel, and overall palatability of products such as emulsified meats, sausages, and salad dressing (Samard and Ryu 2019). Together, these properties affect product formulation, processing behavior, and overall consumer acceptability, making them essential parameters for evaluating the functional performance of food ingredients. The WHC and OHC values of the locust powders are presented in Table 4. WHC values of infrared dried samples ranged from 2.075 to 2.266 g water/g. These values fall within the range reported in previous studies for insect-based powders (Zielińska et al. 2018). The freeze-dried locust powder exhibited a considerably higher WHC value (3.349 g water/g), exceeding those reported by Zielińska et al. (2018) for freeze-dried *S. gregaria* flour (2.18 g water/g) and by Aguilera et al. (2021) for freeze-dried *L. migratoria* flour (2.80 mL water/g). These differences may be attributed to variations in the composition and structural characteristics of the powders (Zielińska et al. 2018). Freeze drying resulted in significantly higher WHC compared to infrared drying. This is likely due to the more porous structure of the freeze-dried locust powder. As water is removed in the frozen state through sublimation, freeze-drying tends to preserve the material’s microstructure with minimal shrinkage, promoting greater water retention (Nowak and Jakubczyk 2020). This finding is consistent with the results of Lucas-González et al. (2019), who reported WHC values of 3.82 and 2.25 g water/g for freeze-dried and oven-dried (60 °C) cricket flour, respectively. Infrared drying temperature did not significantly influence either WHC or OHC values. The OHC of locust powders ranged between 1.584 and 1.707 g oil/g, aligning with previously reported values for insect powders, which vary widely from 77 to 282% depending on the species and processing method (Can Karaca et al. 2023). Zielińska et al. (2018) reported an OHC of 1.98 g oil/g for freeze-dried locust (*S. gregaria*) flour. In the current study, no statistically significant differences were observed in OHC values between the freeze-dried and infrared-dried samples.

## Conclusion

Infrared drying was employed for the production of locust powder. Increasing the drying temperature from 60 to 80 °C significantly enhanced the effective moisture diffusivity (Deff) and resulted in 2.8-fold reduction in drying time. Among the tested models, the Page model was identified as the most suitable for describing the thin layer drying kinetics of locusts. The water activity of locust powders was below the threshold for microbial growth, ensuring product stability and safety during storage. Higher infrared drying temperatures resulted in lower water activity values. The physicochemical and techno-functional properties of infrared-dried locust powders were found to be comparable to those of freeze-dried powder. While freeze-drying yielded the highest water holding capacity (WHC), it did not offer significant advantages over infrared drying in terms of bulk density, flowability, oil holding capacity (OHC), or color parameters. Locust powder exhibited poor flowability, which may pose challenges during manufacturing. Therefore, improving the flow properties is essential for the effective incorporation of locust powder into food production systems. Notably, varying the infrared drying temperature did not lead to a significant change in pH, bulk density, flowability, WHC, or OHC.

This study provides new insights into the drying kinetics of *Locusta migratoria* under infrared conditions and examines its effects on selected physical and functional properties relevant to food powder applications. Infrared drying demonstrated a clear advantage over freeze drying by substantially reducing processing time. Except for WHC, infrared-dried powders exhibited comparable properties to freeze-dried powders, which are often considered the benchmark in terms of quality. Notably, infrared drying has been recognized in the literature as a scalable and energy-efficient technique in the food industry. Considering both its operational efficiency and the favorable characteristics retained in the final product, the findings of this study support the potential integration of infrared drying into commercial insect powder production workflows.

## Data Availability

The datasets used and/or analyzed during the current study are available from the corresponding author on reasonable request.

## Abbreviations

CI: Carr’s index
HR: Hausner ratio
MR: Moisture ratio
OHC: Oil holding capacity
WHC: Water holding capacity

## References

[CR1] Aguilera Y, Pastrana I, Rebollo-Hernanz M, Benitez V, Álvarez-Rivera G, Viejo JL, Martín-Cabrejas MA (2021) Investigating edible insects as a sustainable food source: nutritional value and techno-functional and physiological properties. Food Funct 12:6309–6322. 10.1039/D0FO03291C 34085683 10.1039/d0fo03291c

[CR2] Alsaed AK, Ahmad R, Aldoomy H, El-Qader SA, Saleh D, Sakejha H, Mustafa L (2013) Characterization, concentration and utilization of sweet and acid whey. Pak J Nutr 12:172–177. 10.3923/pjn.2013.172.177

[CR3] Álvarez S, Álvarez C, Hamill R, Mullen AM, O’Neill E (2021) Drying dynamics of meat highlighting areas of relevance to dry-aging of beef. Compr Rev Food Sci Food Saf 20:5370–5392. 10.1111/1541-4337.12845 34601801 10.1111/1541-4337.12845

[CR4] Aykın Dinçer E (2023) Dried meat products obtained by different methods from past to present. Food Rev Int 39:2457–2476. 10.1080/87559129.2021.1956944

[CR5] Azzollini D, Derossi A, Severini C (2016) Understanding the drying kinetic and hygroscopic behaviour of larvae of yellow mealworm (*Tenebrio molitor*) and the effects on their quality. J Insects Food Feed 2:233–243. 10.3920/JIFF2016.0001

[CR6] Barutçu Mazı I (2024) Comparative analysis of nutritional quality and color properties of flours derived from *Locusta migratoria* at different developmental stages. Food Sci Technol Int. 10.1177/10820132241254 10.1177/10820132241254976 38751138

[CR7] Bawa M, Songsermpong S, Kaewtapee C, Chanput W (2020) Effects of microwave and hot air oven drying on the nutritional, microbiological load, and color parameters of the house crickets (*Acheta domesticus*). J Food Process Preserv 44:e14407. 10.1111/jfpp.14407

[CR8] Brennan JG (1994) Food dehydration: a dictionary and guide. Butterworth-Heinemann, Oxford

[CR9] Can Karaca A, Nickerson M, Caggia C, Randazzo CL, Balange AK et al (2023) Nutritional and functional properties of novel protein sources. Food Rev Int 39:6045–6077. 10.1080/87559129.2022.2067174

[CR10] Carr RL (1965) Evaluating flow properties of solids. Chem Eng 72:163–168

[CR11] Chang KS, Kim DW, Kim SS, Jung MY (1998) Bulk flow properties of model food powder at different water activity. Int J Food Prop 1:45–55. 10.1080/10942919809524564

[CR12] Cuq B, Berthiaux H, Gatumel C (2013) Powder mixing in the production of food powders. In: Bhandari B, Bansal N, Zhang M, Schuck P (eds) Handbook of food powders. Woodhead Publishing Limited, Philadelphia, pp 200–229

[CR13] Delfiya DA, Prashob K, Murali S, Alfiya PV, Samuel MP, Pandiselvam R (2022) Drying kinetics of food materials in infrared radiation drying: a review. J Food Process Eng 45:e13810. 10.1111/jfpe.13810

[CR14] Derossi A, Severini C, Cassi D (2011) Mass transfer mechanisms during dehydration of vegetable food: traditional and innovative approaches. In: El-Amin (ed) Advanced topics in mass transfer. InTech-Open Access Publisher, pp 305–354

[CR15] Dutta D, Chaudhuri UR, Chakraborty R (2005) Structure, health benefits, antioxidant property and processing and storage of carotenoids. Afr J Biotechnol 4:1510–1520. 10.4314/ajfand.v4i13.71773

[CR16] Elmas F, Bodruk A, Köprüalan Ö, Arıkaya Ş, Koca N et al (2020) Drying kinetics behavior of turkey breast meat in different drying methods. J Food Process Eng 43:e13487. 10.1111/jfpe.13487

[CR17] Ertekin C, Firat MZ (2017) A comprehensive review of thin layer drying models used in agricultural products. Crit Rev Food Sci Nutr 57:701–717. 10.1080/10408398.2014.910493 25751069 10.1080/10408398.2014.910493

[CR18] Farahmandfar R, Tirgarian B, Dehghan B, Nemati A (2019) Comparison of different drying methods on bitter orange (*Citrus aurantium* L.) peel waste: changes in physical (density and color) and essential oil (yield, composition, antioxidant and antibacterial) properties of powders. J Food Meas Charact 14:862–875. 10.1007/s11694-019-00334-x

[CR19] Fombong FT, Van Der Borght M, Vanden Broeck J (2017) Influence of freeze-drying and oven-drying post blanching on the nutrient composition of the edible insect *Ruspolia differens*. InSects 8:102. 10.3390/insects8030102 28926949 10.3390/insects8030102 PMC5620722

[CR20] Fombong FT, Kinyuru J, Ng’ang’a J, Ayieko M, Tanga CM, Vanden Broeck J, Van Der Borght M (2021) Affordable processing of edible orthopterans provides a highly nutritive source of food ingredients. Foods 10:144. 10.3390/foods10010144 33445608 10.3390/foods10010144 PMC7826988

[CR21] Ganesan V, Rosentrater KA, Muthukumarappan K (2008) Flowability and handling characteristics of bulk solids and powders—a review with implications for DDGS. Biosyst Eng 101:425–435. 10.1016/j.biosystemseng.2008.09.008

[CR22] Goodwin TW (1949) Carotenoid distribution in solitary and gregarious phases of the African migratory locust (*Locusta migratoria migratorioides* R. & F.) and the desert locust (*Schistocerca gregaria* Forsk.). Biochem J 45:472–479. 10.1042/bj0450472 15394441 10.1042/bj0450472 PMC1275031

[CR23] Hausner HH (1967) Friction conditions in a mass of metal powder. Int J Powder Metall 3:7–13

[CR24] Hernández-Álvarez AJ, Mondor M, Piña-Domínguez IA, Sánchez-Velázquez OA, Melgar Lalanne G (2021) Drying technologies for edible insects and their derived ingredients. Dry Technol 39:1991–2009. 10.1080/07373937.2021.1915796

[CR25] Hong S, Shen Y, Li Y (2022) Physicochemical, functional properties of texturized vegetable proteins cooked patty textures: comprehensive, characterization correlation, analysis. Foods 11:2619. 10.3390/foods11172619 36076805 10.3390/foods11172619 PMC9455741

[CR26] Huang D, Yan P, Tang X, Luo L, Sunden B (2021) Application of infrared radiation in the drying of food products. Trends Food Sci Technol 110:765–777. 10.1016/j.tifs.2021.02.039

[CR27] Hutchings JB (1999) Food colour and appearance, 2nd edn. Aspen Publishers, Gaithersburg

[CR28] Ingrisch S, Rentz DCF (2009) Orthoptera: grasshoppers, locusts, katydids, crickets. In: Resh VH, Carde RT (eds) Encyclopedia of insects, 2nd edn. Academic Press, pp 732–743

[CR29] Jakobson K, Kaleda A, Adra K, Tammik ML, Vaikma H, Kriščiunaite T, Vilu R (2023) Techno-functional and sensory characterization of commercial plant protein powders. Foods 12:2805. 10.3390/foods12142805 37509897 10.3390/foods12142805 PMC10379337

[CR30] Kamiloğlu A, Elbir T, Dölek M, Eşrefoğlu ES (2023) Comparative evaluation of different drying techniques for beef meat. J Food Process Eng. 10.1111/jfpe.14441

[CR31] Keil C, Grebenteuch S, Kröncke N, Kulow F, Pfeif S, Kanzler C et al (2022) Systematic studies on the antioxidant capacity and volatile compound profile of yellow mealworm larvae (*T. molitor* L) under different drying regimes. Insects 13:166. 10.3390/insects13020166 35206739 10.3390/insects13020166 PMC8876196

[CR32] Khampakool A, Soisungwan S, You S, Park SH (2020) Infrared assisted freeze-drying (IRAFD) to produce shelf-stable insect food from *Protaetia brevitarsis* (white-spotted flower chafer) larva. Food Sci Anim Resour 40:813–830. 10.5851/kosfa.2020.e60 32968732 10.5851/kosfa.2020.e60 PMC7492168

[CR33] Khatun H, Claes J, Smets R, De Winne A, Akhtaruzzaman M, Van Der Borght M (2021) Characterization of freeze-dried, oven-dried and blanched house crickets (*Acheta domesticus*) and Jamaican field crickets (*Gryllus assimilis*) by means of their physicochemical properties and volatile compounds. Eur Food Res Technol 247:1291–1305. 10.1007/s00217-021-03709-x

[CR34] Koç M, Koç B, Güngör Ö, Ertekin FK (2012) The effects of moisture on physical properties of spray-dried egg powder. Dry Technol 30:567–573. 10.1080/07373937.2011.651546

[CR35] Koç B, Koç M, Baysan U (2021) Food powders bulk properties. In: Ermiş E (ed) Food powders properties and characterization. Food engineering series. Springer, Cham, pp 1–36

[CR36] Kröncke N, Böschen V, Woyzichovski J, Demtröder S, Benning R (2018) Comparison of suitable drying processes for mealworms (*Tenebrio molitor*). Innov Food Sci Emerg Technol 50:20–25. 10.1016/j.ifset.2018.10.009

[CR64] Liguori B, Sancho AI, Poulsen M, Lindholm Bøgh K (2022) Novel foods: allergenicity assessment of insect proteins. EFSA J 20:e200910. 10.2903/j.efsa.2022.e200910 36531270 10.2903/j.efsa.2022.e200910 PMC9749447

[CR37] Liu Y, Zhang Z, Hu L (2022) High efficient freeze-drying technology in food industry. Crit Rev Food Sci Nutr 62:3370–3388. 10.1080/10408398.2020.1865261 33393368 10.1080/10408398.2020.1865261

[CR38] López-Malo A, Alzamora SM (2015) Water activity and microorganism control: past and future. In: Gutiérrez-López GF, Alamilla-Beltrá L, del Pilar BM, Welti-Chanes J, Parada-Arias E, Barbosa-Cánovas GV (eds) Water stress in biological, chemical, pharmaceutical and food systems. Springer, New York, pp 245–262

[CR39] Lucas-González R, Fernández-López J, Pérez-Álvarez JA, Viuda-Martos M (2019) Effect of drying processes in the chemical, physico-chemical, techno-functional and antioxidant properties of flours obtained from house cricket (*Acheta domesticus*). Eur Food Res Technol 245:1451–1458. 10.1007/s00217-019-03301-4

[CR40] Lumay G, Boschini F, Traina K, Bontempi S, Remy JC, Cloots R, Vandewalle N (2012) Measuring the flowing properties of powders and grains. Powder Technol 224:19–27. 10.1016/j.powtec.2012.02.015

[CR41] Melgar-Lalanne G, Hernández-Álvarez AJ, Salinas-Castro A (2019) Edible insects processing: traditional and innovative technologies. Compr Rev Food Sci Food Saf 18:1166–1191. 10.1111/1541-4337.12463 33336989 10.1111/1541-4337.12463

[CR42] Menozzi D, Sogari G, Veneziani M, Simoni E, Mora C (2017) Eating novel foods: an application of the theory of planned behaviour to predict the consumption of an insect-based product. Food Qual Prefer 59:27–34. 10.1016/j.foodqual.2017.02.001

[CR43] Mishyna M, Haber M, Benjamin O, Martinez JI, Chen J (2020) Drying methods differentially alter volatile profiles of edible locusts and silkworms. J Insects Food Feed 6:405–415. 10.3920/JIFF2019.0046

[CR44] Mishyna M, Keppler JK, Chen J (2021) Techno-functional properties of edible insect proteins and effects of processing. Curr Opin Colloid Interface Sci 56:101508. 10.1016/j.cocis.2021.101508

[CR65] Morr CV, German B, Kinsella JE, Regenstein JM, Buren JV, Kilara A, Lewis BA, Mangino ME (1985) A collaborative study to develop a standardized food protein solubility procedure. J Food Sci 50(6):1715–1718. 10.1111/j.1365-2621.1985.tb10572.x

[CR45] Mugova AK, Zvidzai CJ, Musundire R (2021) Techno-functional properties of armoured cricket (*Acanthoplus discoidalis*) flour. Sch J Eng Tech 8:102–112. 10.36347/sjet.2021.v09i08.001

[CR46] Niamnuy C, Devahastin S, Soponronnarit S, Vijaya Raghavan GS (2008) Kinetics of astaxanthin degradation and color changes of dried shrimp during storage. J Food Eng 87:591–600. 10.1016/j.jfoodeng.2008.01.013

[CR47] Nowak D, Jakubczyk E (2020) The freeze-drying of foods-the characteristic of the process course and the effect of its parameters on the physical properties of food materials. Foods 9:1488. 10.3390/foods9101488 33080983 10.3390/foods9101488 PMC7603155

[CR48] Nyangena DN, Mutungi C, Imathiu S, Kinyuru J, Affognon H, Ekesi S et al (2020) Effects of traditional processing techniques on the nutritional and microbiological quality of four edible insect species used for food and feed in East Africa. Foods 9:574. 10.3390/foods9050574 32375385 10.3390/foods9050574 PMC7278588

[CR49] Omuse ER, Tonnang HE, Yusuf AA, Machekano H, Egonyu JP et al (2024) The global atlas of edible insects: analysis of diversity and commonality contributing to food systems and sustainability. Sci Rep 14:5045. 10.1038/s41598-024-55603-7 38424443 10.1038/s41598-024-55603-7 PMC10904393

[CR50] Ozyalcin ZO, Kipcak AS, Tugrul N (2023) The effect of various methods on the drying kinetics and mathematical modelling of seabass (*Dicentrarchus labrax*). J Aquat Food Prod Technol 32:384–395. 10.1080/10498850.2023.2227853

[CR51] Parniakov O, Mikhrovska M, Wiktor A, Alles M, Ristic D et al (2022) Insect processing for food and feed: a review of drying methods. Dry Technol 40:1500–1513. 10.1080/07373937.2021.1962905

[CR52] Rajpurohit B, Chen G, Li Y (2025) Water and oil holding capacity. In: Li Y (ed) Plant-based proteins. Humana, New York, pp 335–344

[CR53] Rockland LB, Stewart GF (2013) Water activity: influences on food quality: a treatise on the influence of bound and free water on the quality and stability of foods and other natural products. Academic Press, New York

[CR54] Romdhane NG, Bonazzi C, Kechaou N, Mihoubi NB (2015) Effect of air-drying temperature on kinetics of quality attributes of lemon (*Citrus limon* cv. lunari) peels. Dry Technol 33:1581–1589. 10.1080/07373937.2015.1012266

[CR55] Saker A, Cares-Pacheco MG, Marchal P, Falk VJPT (2019) Powders flowability assessment in granular compaction: what about the consistency of Hausner ratio? Powder Technol 354:52–63. 10.1016/j.powtec.2019.05.032

[CR56] Samard S, Ryu GH (2019) Physicochemical and functional characteristics of plant protein-based meat analogs. J Food Process Preserv 43:e14123. 10.1111/jfpp.14123

[CR57] Shenoy P, Viau M, Tammel K, Innings F, Fitzpatrick J, Ahrné L (2015) Effect of powder densities, particle size and shape on mixture quality of binary food powder mixtures. Powder Technol 272:165–172. 10.1016/j.powtec.2014.11.023

[CR58] Simpson R, Ramírez C, Nuñez H, Jaques A, Almonacid S (2017) Understanding the success of Page’s model and related empirical equations in fitting experimental data of diffusion phenomena in food matrices. Trends Food Sci Technol 62:194–201. 10.1016/j.tifs.2017.01.003

[CR59] Singh Y, Cullere M, Kovitvadhi A, Chundang P, Dalle Zotte A (2020) Effect of different killing methods on physicochemical traits, nutritional characteristics, in vitro human digestibility and oxidative stability during storage of the house cricket (*Acheta domesticus* L.). Innov Food Sci Emerg Technol 65:102444. 10.1016/j.ifset.2020.102444

[CR60] Son YJ, Lee JC, Hwang IK, Nho CW, Kim SH (2019) Physicochemical properties of mealworm (*Tenebrio molitor*) powders manufactured by different industrial processes. LWT 116:108514. 10.1016/j.lwt.2019.108514

[CR61] Suhag R, Kelli A, Razem M (2024) Factors influencing food powder flowability. Powders 3:65–76. 10.3390/powders3010006

[CR62] Van Huis A (2017) Did early humans consume insects? J Insects Food Feed 3:161–163. 10.3920/JIFF2017.x006

[CR63] Zielińska E, Karaś M, Baraniak B (2018) Comparison of functional properties of edible insects and protein preparations thereof. LWT 91:168–174. 10.1016/j.lwt.2018.01.058

